# Supplementary material for: Exploiting Chromosomal Instability of PTEN-Deficient Triple-Negative Breast Cancer Cell Lines for the Sensitization Against PARP1 Inhibition in a Replication-Dependent Manner
Source: Cancers (Basel). 2020 Sep 29;12(10):2809. doi: 10.3390/cancers12102809 (PMC7601067; doi:10.3390/cancers12102809)
Supplement: Supplementary file 1 [file cancers-12-02809-s001.pdf]

cancers

MDPI

Supplementary Material: Exploiting Chromosomal Instability of PTEN 3 Deficient Triple Negative Breast Cancer Cell Lines for 4 the Sensitization Against PARP1 Inhibition in a 5 Replication Dependent Manner

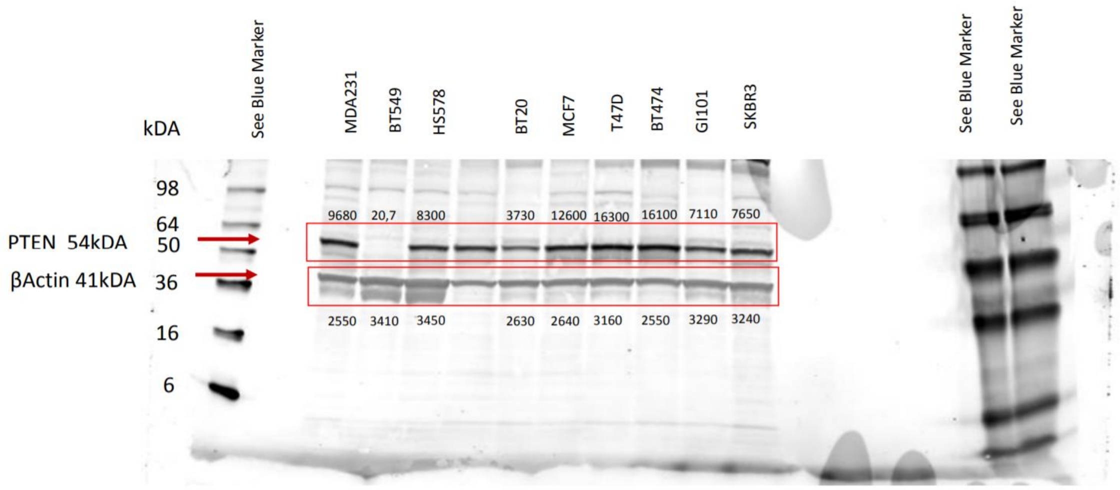

Figure S1. Raw data of Western blot from figure 1 (**b**) Numbers above and below the box indicate the original densitometry values.

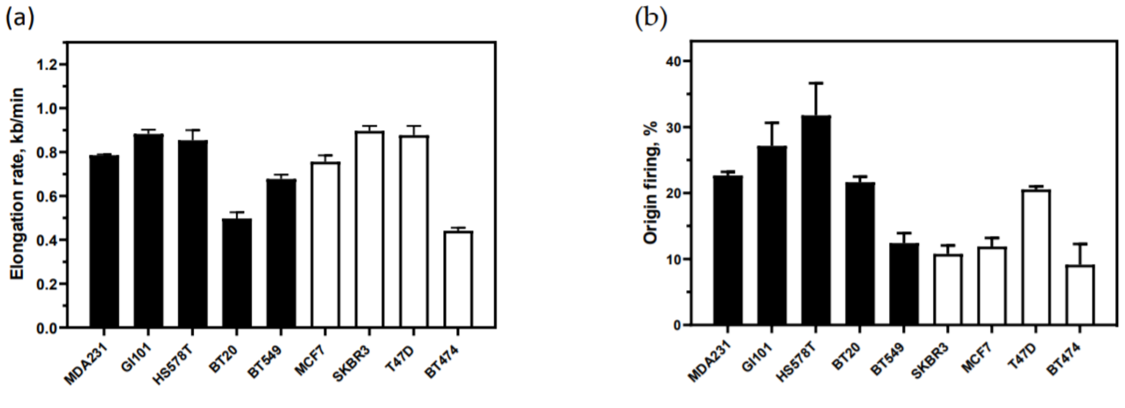

Figure S2. Raw data figure 2 (**a**) for elongation rate, (**b**) for origin firing.

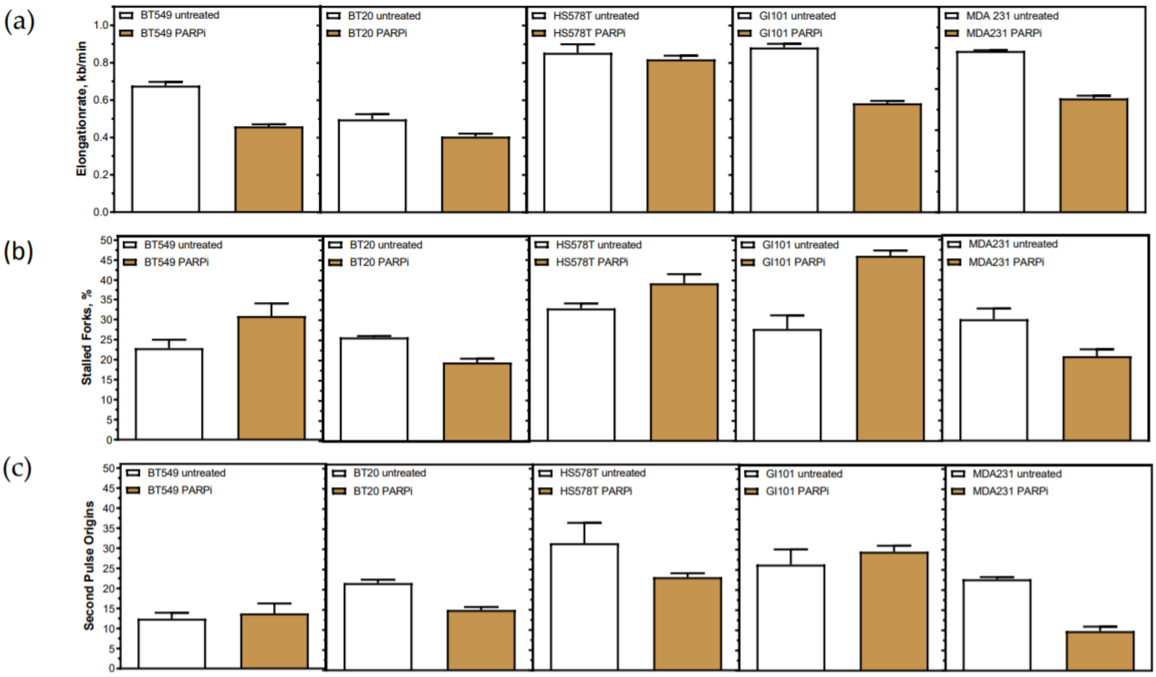

Figure S3. Raw data from DNA fiber analysis. (**a**) Raw data for figure 3e elongation tract length, (**b**) Raw data for figure 3f replication fork stalling and (**c**) Raw data for figure 3g new origin firing after PARP1 inhibition.

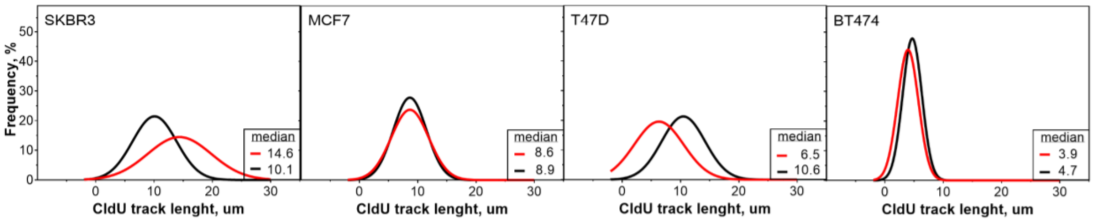

Figure S4. Fork instability after HU treatment in SKBR3, MCF7, T47D and BT474.

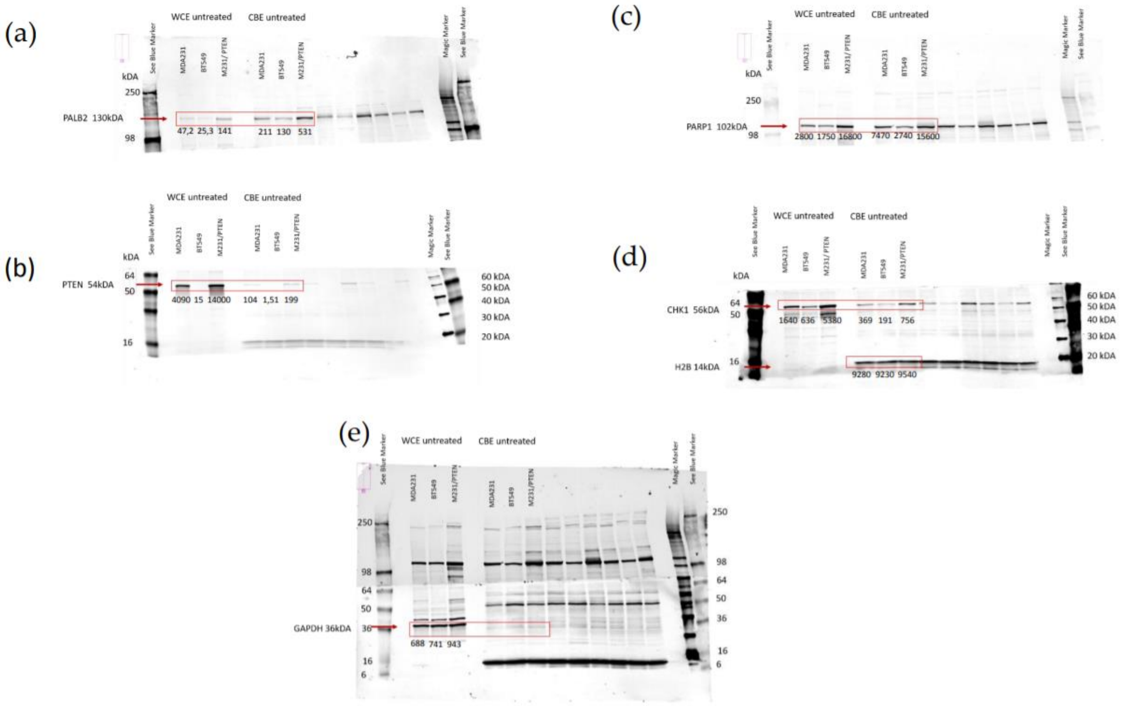

Figure S5. Raw data of Western blot from figure 6 (**a**), Numbers below the boxes indicate the original densitometry values.

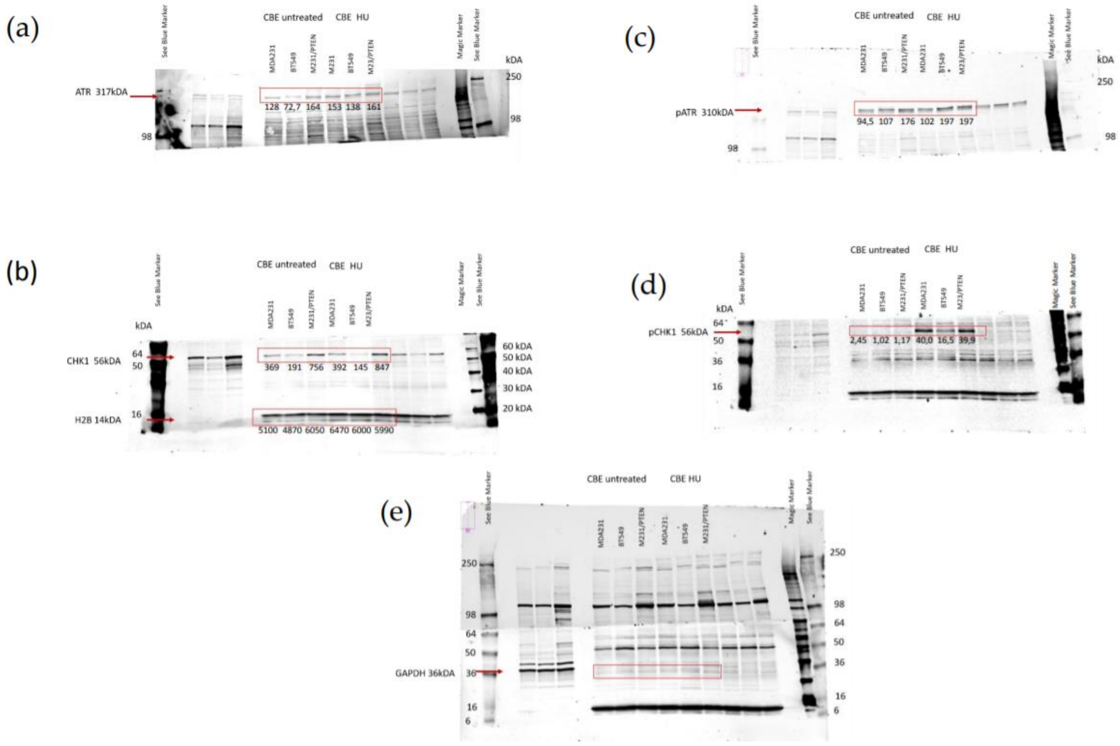

Figure S6. Raw data of Western blot from figure 6 (**b**), Numbers below the boxes indicate the original densitometry values.

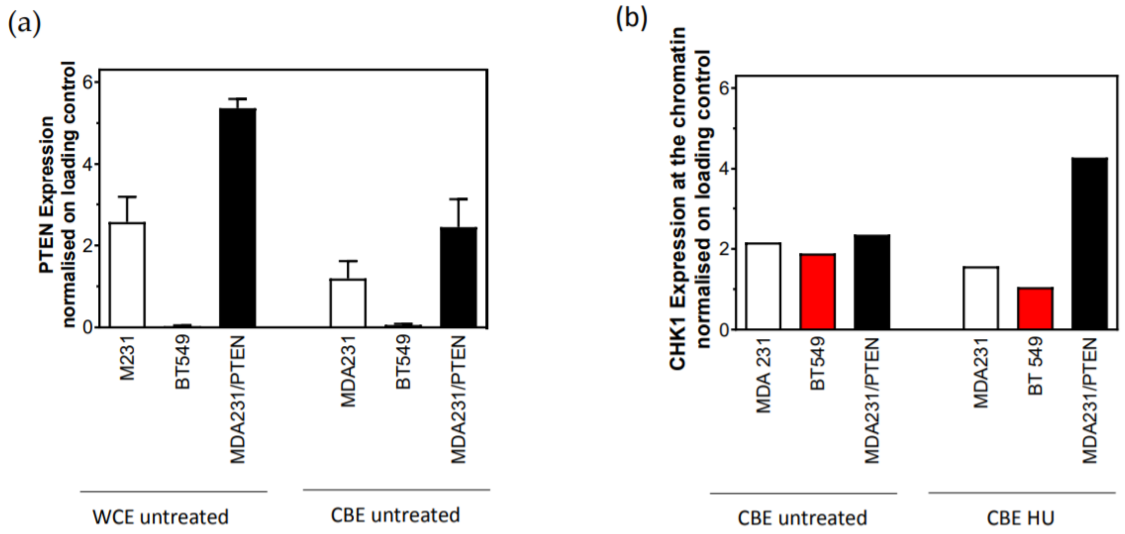

Figure S7. PTEN expression and recruitment of DNA repair proteins to the chromatin in TNBC. (**a**) Quantitative analysis of PTEN expression in whole cell extracts (WCE) and chromatin bound extracts of fractionated cell extracts (CBE) of untreated TNBC cell lines, normalized on loading control. (**b**) Quantitative analysis of CHK1 expression in CBE after treatment with 2mM HU for four hours in comparison to untreated controls in TNBC cell lines, normalized on loading control.

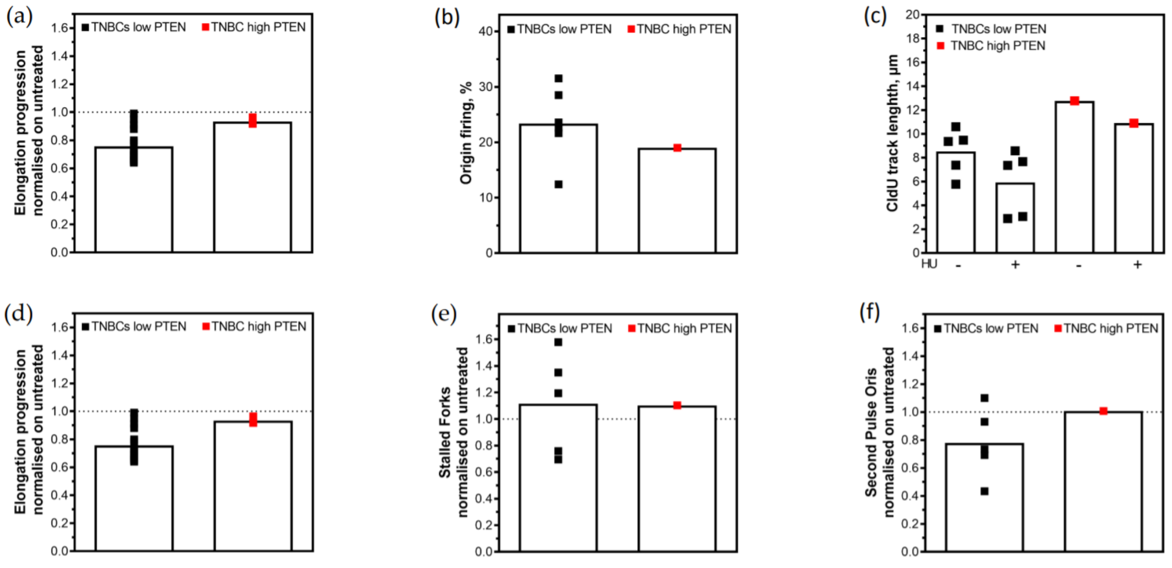

Figure S8. Comparison of TNBCs with low PTEN expression to TNBC with high PTEN expression in replication fork elongation, stalling and new origin firing untreated and upon PARP1 inhibition.

© 2020 by the authors. Submitted for possible open access publication under the terms and conditions of the Creative Commons Attribution (CC BY) license (http://creativecommons.org/licenses/by/4.0/).
